# Optimizing Medication Use in Older Adults in Primary Care: A Systematic Review of the Effectiveness of Deprescribing Interventions

**DOI:** 10.1111/ggi.70677

**Published:** 2026-07-23

**Authors:** Emanuel Miranda Oliveira, Luan Carrijo Ferreira, Mateus Caetano Silva, Wallisen Tadashi Hattori

**Affiliations:** ^1^ Department of Public Health, School of Medicine Federal University of Uberlândia Uberlândia Brazil; ^2^ School of Medicine Federal University of Uberlândia Brazil

**Keywords:** deprescribing, older adults, polypharmacy, potentially inappropriate medications, primary health care

## Abstract

**Objective:**

The increasing prevalence of polypharmacy and potentially inappropriate medications (PIMs) among older adults in Primary Health Care (PHC) poses significant risks to older patients. However, the best deprescribing approach is not well established. This systematic review aimed to evaluate the effectiveness and applicability of various deprescribing techniques in PHC.

**Methods:**

We conducted a systematic review of randomized clinical trials from 2010 to 2026 across the PubMed, EMBASE, and LILACS databases that compared deprescribing techniques with usual care in older patients in PHC. A narrative synthesis was employed to present the findings, and the quality of included studies was rigorously assessed using the Cochrane Risk of Bias Tool (RoB 2) and the GRADE system.

**Results:**

From a screening of 1032 unique citations, 17 studies met the inclusion criteria. These studies investigated a range of deprescribing interventions, including automated algorithms, multidisciplinary approaches, educational activities, pharmacist‐led, and physician‐conducted medication reviews. Automated algorithms and pharmacist‐led interventions consistently demonstrated significant efficacy in reducing potentially inappropriate medications. Conversely, multidisciplinary interventions and educational activities yielded mixed results regarding overall medication reduction. Notably, medication reviews performed solely by family physicians did not reduce PIMs. No significant effects on mortality were observed across any interventions, and a cluster‐level reduction in hospitalizations was reported by one study.

**Conclusions:**

Deprescribing techniques, particularly automated algorithms and pharmacist‐led interventions, appear effective in reducing the use of potentially inappropriate medications among older adults in PHC. Future research should prioritize standardizing outcomes and conducting studies designed to comprehensively assess the clinical impacts of deprescribing.

## Graphical abstract

Automated algorithms and pharmacist‐led reviews are most effective at reducing PIMs, with no increased harm observed. No significant mortality impact was found. Hospital admission evidence is limited—one study showed a practice‐level reduction as a secondary outcome, requiring cautious interpretation.

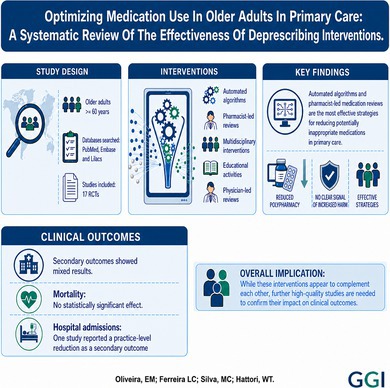

## 1 Introduction

The use of multiple medicines (or polypharmacy) represents a critical health issue in the older population. One‐third of individuals aged 65 or older regularly use 5 or more medications [1]. Among these, some pose a higher risk of adverse clinical outcomes than potential benefits, or have safer and more effective alternatives, and are therefore classified as Potentially Inappropriate Medications (PIMs) [2]. Some authors expand the concept to Potentially Inappropriate Prescribing (PIP), when medications lack a clear clinical indication, involve an incorrect dose, frequency, or modality of administration, or involve an incorrect duration of treatment, or the omission of a clinically indicated medication [3]. The pooled prevalence of PIM use across 17 countries was 36.7%, with a rising trend over the past two decades [4]. The prevalence of PIM varies significantly in the literature, often depending on the specific criteria used (e.g., Beer's Criteria, STOPP/START criteria, explicit vs. implicit tools). Furthermore, PIM use in this population raises healthcare costs [5] and is a significant cause of emergency department visits [6]. Early detection and discontinuation of PIMs can prevent adverse drug events and improve the care and quality of life of older adults [7].

Deprescribing is a structured and supervised process of tapering or discontinuing medications that may cause harm or provide no clear benefit [8]. Either a criterion‐based (explicit) or a clinical judgment‐based (implicit) approach can be used to assess prescription appropriateness [9]. This process can be conducted across different healthcare settings, but Primary Health Care (PHC) is particularly relevant given its role in first‐contact care and longitudinal follow‐up of older patients. Therefore, a comparative analysis of deprescribing techniques in PHC is warranted.

Thus, this study aims to evaluate the effectiveness and applicability of various deprescribing techniques for older adults across different PHC settings to reduce long‐term medication use.

## 2 Methods

Our protocol was registered in the International Prospective Register of Systematic Reviews (PROSPERO) number CRD42023416481. The review strictly followed the Preferred Reporting Items for Systematic Reviews and Meta‐Analyses (PRISMA) 2020 [10] guidelines for systematic review reporting.

### 2.1 Inclusion and Exclusion Criteria

**FIGURE 1:**
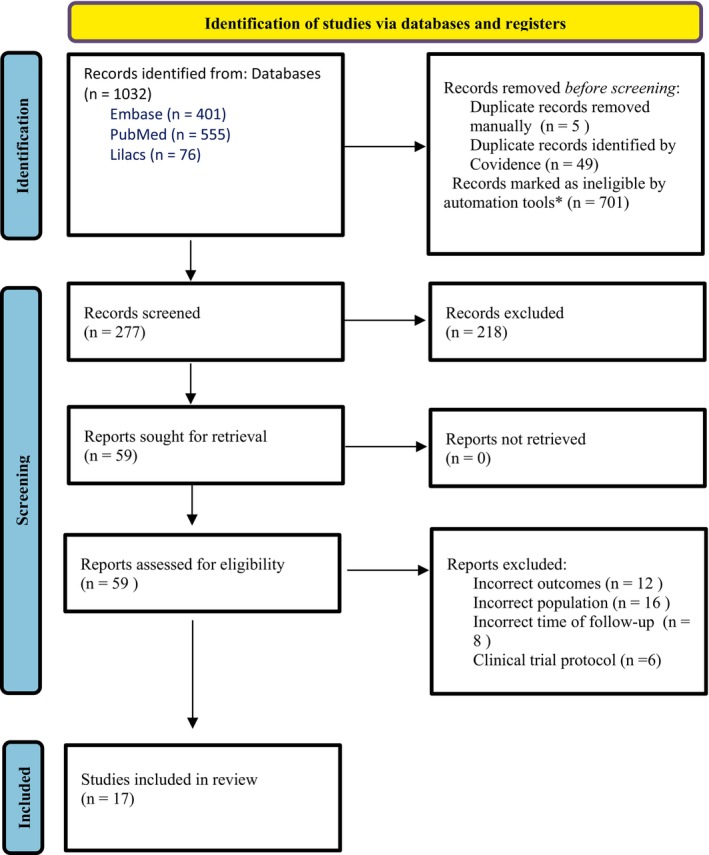
Prisma 2020 flow diagram of study screening and selection. *An additional exclusion was performed using the advanced search options in PubMed, Embase and Lilacs databases. Specific filters applied are in the Appendix S1.

Participants included individuals aged 60 years or older, including patients from developing countries, residing at home or in long‐term care facilities, with or without polypharmacy, cognitive impairment, or multimorbidity, who received care in PHC settings; palliative or end‐of‐life cases were excluded to focus on the general older adult population. Interventions were structured deprescribing techniques. Comparators were usual care without specific deprescribing interventions. Primary outcomes focused on reductions in long‐term medication use (6 months or more), decreases in PIMs, or resolution of PIP, with secondary outcomes including changes in hospital admissions and mortality rates. The timeframe spanned studies published from 2010 to 2026, with emphasis on contemporary, robust PHC‐relevant trials. Study designs were restricted to randomized controlled trials (RCTs) and cluster‐randomized controlled trials (cRCTs) to provide high‐quality evidence (Figure 1).

### 2.2 Search Strategy

Our search strategy (Appendix S1) was meticulously developed to include terms related to deprescribing techniques and covered the following databases: PubMed, EMBASE, and LILACS. Searches were conducted on February 28, 2026. A specialized librarian assisted in refining and optimizing the search terms.

### 2.3 Data Extraction and Quality Assessment

Initial screening of titles and abstracts was independently conducted by two researchers, one of whom was a physician. The full‐text assessments were performed by two researchers. Data extraction was carried out by one researcher and reviewed by another, including a physician. We used Covidence [11] systematic review software. Study quality was assessed using the Cochrane Risk of Bias Tool (RoB 2) [12], and the certainty of the evidence was evaluated using the Grading of Recommendations Assessment, Development and Evaluation (GRADE) [13] system. For each step, discrepancies were resolved by consensus and, if necessary, consultation with a third reviewer.

### 2.4 Synthesis and Analysis

We conducted a narrative synthesis of the findings following the Synthesis Without Meta‐analysis (SWiM) [14] protocol. For each included study, we recorded publication year, study design, country, description of the deprescribing technique, sample size, mean participant age, and primary and secondary outcomes (mortality and hospital admissions).

## 3 Results

The initial search found 1032 records. Covidence removed 54 duplicates, and automated tools removed 701 records. We screened 277 unique records by title and abstract. Of these, 218 were ineligible. Full‐text analysis of 59 records led to 36 exclusions. We included 17 studies in the review (Figure 1).

The mean participant age ranged from 69.6 to 87.0 years. Participants either lived at home or resided in long‐term care facilities and received the intervention in PHC units.

The included studies described several deprescribing techniques: clinical decision‐support tools with automated algorithms, complex multidisciplinary interventions, educational activities for physicians and/or patients, and prescription reviews by pharmacists or family physicians. Control groups received routine PHC care, physician reassessment with medication renewal, or medication use review. Table 1 summarizes the results.

**TABLE 1:** Deprescribing techniques.

Authors [References]	Study design	Country	Technique	Controls	*n*	Primary outcome	Death	Hospital admission	Follow‐up (months)	Additional information
Automated algorithm of deprescribing recommendations										
Clyne et al. [15]	cRCT	Ireland	Medication review using a web‐based pharmaceutical treatment algorithm, indicating why the prescription may be inappropriate, alternative treatment options, and other relevant information.	Usual care. Prescription on a monthly or 3‐month basis. Submission of a list summarizing the medication.	196 C: 97/I: 99	Patients having PIP: OR = 0.32 (0.15–0.70) *p* = 0.02	N/P	N/P	6	The mean PIP was also evaluated. MD = −0.48 (−0.80 to −0.17) *p* = 0.02 The greatest reduction was in proton pump inhibitor, the most used PIM in the study population.
Rieckert et al. [16]	cRCT	Austria United Kingdom Germany Italy	Use of the PRIMA‐eDS system. The tool analyzes diagnoses, medications in use, symptoms, anthropometric measurements, and laboratory tests with recommendations for suspension or replacement of medications.	Usual care, including adhering to any current guidelines, some country‐specific but without using the PRIMA‐eDS tool.	3904 C: 1951/I: 1953	Mean number of medications: IRR^a^ = 0.95 (0.94–0.97) *p* < 0.01	OR = 1.01 (0.73–1.38) *p* = 0.96	OR = 0.92 (0.76–1.10) *p* = 0.36	24	The change in the total number of medications was also evaluated. MD = −0.45 (−0.63 to −0.26) *p* < 0.001
McCarthy et al. [17]	cRCT	Ireland	Use of the SPPiRE tool, a website that reviews medications in use, focusing on deprescribing potentially inappropriate medications and suggesting alternative treatments.	Usual care.	404 C: 196/I: 208	Mean number of medications: AD = 0.95 (0.89–0.99) *p* = 0.04	N/P	N/P	6	The percentage of patients with at least 1 PIP was also evaluated. AD = 0.39 (0.14–1.06) *p* = 0.066 Mean number of PIP. AD = 0.92 (0.81–1.06) *p* = 0.256
Jungo et al. [18]	cRCT	Switzerland	Use of STRIPA tool, an electronic clinical decision support system that analyzes adverse effects, recommendations according to STOPP/START criteria, and appropriate dosage adjusted by renal function. The tool generated recommendations on deprescribing.	Standard unit care with medication review by general practitioner followed by shared decision‐making between practitioner and patient.	323 C: 163/I: 160	Mean number of medications: MD_A_ = 0.0 (−0.87 to 0.97) *p* 0.91 MD_B_ = 0.26 (−0.64 to 1.16) *p* = 0.58	N/P	N/P	A: 6 B: 12	The number of patients with improvement in MAI in 12 months was also evaluated. OR = 1.05 (0.59–1.87) *p* = 0.87
Lauffenburger et al. [19]	cRCT	USA	EHR‐based clinical decision support alerts with information about the risks of ongoing medication use and included a hyperlink to patient discussion points/helpful hints and links to medication activity. It also offers alternative treatments, lifestyle modification handouts, referrals, and pre‐set tapering algorithms tailored to patients' medications and dosages.	Usual care.	3063 C: 1204/I: 1859	Primary Composite Outcome Best Arm: OR = 1.54 (1.01–2.35) *p* = 0.04	N/P	N/P	12	In the primary analyses, none of the behavioral science factors significantly improved deprescribing compared with arms without that factor. Secondary findings: Open encounter timing performed better than order entry timing: OR = 1.25 (1.01–1.56) *p* < 0.05 Pre‐commitment perform better within Open Encounter arms: OR = 1.48 (1.03–2.12) *p* = 0.03
Multifaceted and/or multidisciplinary intervention										
Romskaug et al. [20]	cRCT	Norway	Geriatric assessment and collaborative medication reviews by geriatrician and family physician and clinical follow‐up.	Usual care.	174 C: 87/I: 87	MAI: MD = −6.9 (−9.1 to −4.7) *p* ^b^ < 0.01	OR = 0.36 (0.08–1.58)	OR = 2.03 (0.98–4.24)	6	The outcome after 16 weeks was also assessed. MD = −6.5 (−8.6 to −4.3) *p* ^b^ < 0.01
Strauven et al. [21]	cRCT	Belgium	Online educational program associated with interdisciplinary meetings and interdisciplinary case conferences to discuss medication use, with financial incentives for professionals who participate in the case conferences.	Usual care.	1804 C: 957/I: 847	A: At least 1 PIM or omission that had been solved: OR = 2.66 (1.78–3.98) *p* < 0.01 B: No new PIM or omission: OR = 0.66 (0.46–0.96) *p* = 0.03 A + B: OR = 1.48 (1.06–2.06) *p* = 0.02	N/P	N/P	15	Authors considered more than one outcome that reflects deprescribing, and the sum of the outcomes is the primary outcome of the study.
Mahlknecht et al. [22]	cRCT	Italy	Multidisciplinary assessments of medication regimens. The general practitioners were invited to reflect on the recommendations in a shared decision‐making process with the patient.	Usual care. General practitioners could use guidelines. They did not receive a structured review of the medications in use.	579 C: 272/I: 307	Number of medications: median (IQR) CG = 9 (8–10) IG = 8 (7–10) *p* = 0.1	OR = 1.18 (0.51–2.72) *p* = 0.7	OR = 1.39 (0.95–2.03) *p* = 0.09	24	The total number of medications was also evaluated. CG = 2140, IG = 2218 Change in the number of medications at the end of the study. Median (IQR) = 0 (−2 to 0) *p* = 0.6
Toivo et al. [23]	cRCT	Finland	Prescription review by a community pharmacist associated with Drug Related Problem Risk Assessment Tool (DRP‐RAT) by nurse and action plan from DRP‐RAT.	Care was based on the patient's needs, with help in daily activities and medicine use.	129 C: 64/I: 65	Number of medications: AMD = 0.52 (−0.37 to 1.41) *p* = 0.25	N/P	N/P	12	Harmful medication users were also evaluated. AMD = 1 (0.63–1.60) *p* = 1.0 And Beers Criteria medication users. AMD = 0.84 (0.39–1.82) *p* = 0.66
Educational activities										
Zechmann et al. [24]	cRCT	Switzerland	A 2‐h training session on polypharmacy using an adapted Good Palliative‐Geriatric Practice (GPGP) deprescribing algorithm. The physician reviewed the patient's medication list, identified the main diagnoses and complaints, assessed each drug's appropriateness, and shared decision‐making with the patient to stop, reduce, or replace medications.	A general lecture on multimorbidity instructions.	334 C: 206/I: 128	Mean difference of medications: A: −0.47 (−1.07 to 0.12) *p* = 0.12 B: −0.04 (−0.63 to 0.56) *p* = 0.91	**B: RR: 0.87 (0.35–2.11)** ***p* = 0.75**	B: RR: 1.28 (0.92–1.79) *p* = 0.15	A: 6 B: 12	The intervention had a significant immediate effect after the first consultation. −0.85 (−1.38 to −0.32) *p* < 0.01
Rudolf et al. [25]	cRCT	Germany	Training with health professionals according to the need and request. Presentation of a pocket guide based on the Priscus list with 17 medications that should be avoided and three medication interactions and how to properly monitor the use of medicines. The training was performed with physicians alone and with the entire practice team in another group.	Usual recommendations for pharmacotherapy to the elderly. The physicians were invited to perform continuing education about pharmacotherapy. The health team was not involved.	1138 C: 562/I1: 285 I2: 291	Number of patients with at least one PIM or DDI MD^b^ 1 + 2 = −3.7 (−10.1 to 2.7) *p* = 0.26 MD1 = −6.4 (−14.2 to 1.4) *p* = 0.11 MD2 = −1.1 (−9.37 to 7.16) *p* = 0.79	MD^b^ = 1.6 (0.82–4.02) *p* = 0.2	MD^b^ = −8.6 (−14.89 to −2.31) *p* < 0.01	12	Two interventions were performed. In intervention 1, only physicians received the educational activity. In intervention 2, the entire practice team received educational activities. For mortality and hospital admissions, MD was used among PHC clinics.
Bayliss et al. [26]	cRCT	USA	Educational activities performed with patient/caregiver with material distribution on the optimization of medicines. Educational activities with physicians about the optimization of medicines and deprescribing options.	Review of routine medication reconciliation at primary care visits with electronic health record alerts for potentially high‐risk prescribing.	3012 C: 1579/I: 1433	Number of medications: AD = **−0.1** **(−0.2 to 0.04)** ***p* = 0.14**	**OR** ^b^ **= 1.04** **(0.78–1.41)** ***p* = 0.77**	N/P	6	The percentage of patients with at least one PIM was also evaluated. **AD = −3.2** **(−6.2 to 0.4)** ***p* = 0.08** And sensitivity analysis for patients with 90 days or more of follow‐up. **AD = −3.2** **(−6.3 to 0.4)** ***p* = 0.08**
Cura‐Gonzalez et al. [27]	cRCT	Spain	Four‐week course for physicians on an electronic health record system on topics related to multimorbidity, polypharmacy, and deprescribing.	Usual care with guidelines and protocols of each isolated clinical condition.	593 C: 295/I: 298	MAI **AD**_**A**_ **= −2.42** **(−4.27 to −0.50)** ***p* = 0.01** **AD**_**B**_ **= −3.40** **(−5.45 to −1.34)** ***p* = 0.01**	N/P	**AD = −0.14** **(−0.57 to 0.30)** ***p* = 0.52**	A: 6 B: 12	The study showed an increased effect over time.
Jones et al. [28]	cRCT	USA	An educational brochure mailed to eligible patients before their primary care visit, about proton pump inhibitors (PPI), high‐dose gabapentin, and diabetes agents with hypoglycemia risk. Information on deprescribing, tapering options, and encouragement to discuss medication changes with clinicians. PCPs also received an email with study information and deprescribing resources.	Usual care.	4928 C: 2448/I: 2480	PIM was stopped or dose‐reduced: **OR = 1.19 (1.04–1.35)** ***p* = 0.01**	N/P	N/P	12	
Medication review by pharmacists										
Campins et al. [29]	RCT	Spain	A trained and experienced clinical pharmacist evaluated medications using the GP–GP algorithm and decided about appropriateness on the STOPP/START criteria. The pharmacist makes recommendations for each drug with the physician. The recommendations were discussed with the patient, and a final decision was agreed by physicians and their patients.	Usual care.	503 C: 251/I: 252	1: Drug Discontinuation: *N* **A: 4.37 (2.90–6.44)** ***p* < 0.01** **B: 1.85 (1.17–2.90)** ***p* < 0.01** 2: Dose adjustments: *N* **A: 4.08 (2.79–5.97)** ***p* < 0.01** **B: 3.94 (2.70–5.74)** ***p* < 0.01** 3: Drug substitutions: *N* **A: 2.31 (1.58–3.37)** ***p* < 0.01** **B: 1.54 (1.08–2.19)** ***p* = 0.02**	A: RR 4.99 (0.59–42.2) *p* = 0.14 B: 1.18 (0.40–3.46) *p* = 0.76^b^	A: RR: 1.13 (0.71–1.80) *p* = 0.6^b^ B: 1.11 (0.81–1.51) *p* = 0.5^b^	A: 6 B: 12	
Martinez‐Sotelo et al. [30]	cRCT	Spain	Pharmacist‐led medication review with recommendation of treatment options.	No intervention was performed.	549 C: 272/I: 277	Number of PIM per patient: **MD = −0.43** **(−0.54 to −0.32)** ***p* < 0.01**	**OR** ^b^ **= 1.23** **(0.33–4.64)** ***p* = 0.76**	**MDH** ^b^ **= −0.37** **(−1.50 to 0.76)** ***p* = 0.52**	12	The number of patients with one or more PIMs was also estimated. **OR** ^b^ **= 0.41** **(0.27–0.63)** ***p* < 0.01**
Medication review by family physicians										
Rikala et al. [31]	RCT	Finland	Annual medication assessment conducted by physicians with recommendation of changes to patients.	Usual care.	700 C: 339/I: 361	Patients with at least one psychotropic: **A: OR** ^b^ **= 1.29** **(0.93–1.77)** ***p* = 0.12** **B: OR** ^b^ **= 1.19** **(0.93–1.77)** ***p* = 0.12** **C: OR** ^b^ **= 1.06** **(0.75–1.51)** ***p* = 0.71**	N/P	N/P	A: 12 B: 24 C: 36	

Abbreviations: AD, adjusted difference; AMD, adjusted mean difference; CG, control group; cRCT, cluster‐randomized controlled trial; DDI, undesired drug–drug interaction; IG, intervention group; IQR, interquartile range; IRR, incidence rate ratio; MAI, medication appropriateness index; MD, mean difference; MDH, mean difference of hospitalization days and emergency service visits; N/P, not performed; OR, odds ratio; PHC, primary health care; PIM, potentially inappropriate medication; PIP, potentially inappropriate prescribing; RCT, randomized controlled trial.

^a^ Calculated by authors.

^b^ Incidence rate ratio of the number of medications in the intervention group and control group.

*Source:* Authors.

### 3.1 Automated Algorithm of Deprescribing Recommendations

Rieckert et al. [16] reported fewer medications in the deprescribing group (IRR = 0.95, 95% CI: 0.94–0.97, *p* < 0.01). McCarthy et al. [17] found a similar result (AD = 0.95, 95% CI: 0.89–0.99, *p* = 0.04). Clyne et al. [15] showed deprescribing patients had lower odds of PIPs than usual care (OR = 0.32, 95% CI: 0.15–0.70, *p* = 0.02) and fewer mean PIPs (MD = −0.48, 95% CI: −0.80 to −0.17, *p* = 0.02). Lauffenburger et al. [19] showed improvement in discontinuation or tapering (OR = 1.54, 95% CI: 1.01–2.35, *p* = 0.04).

Jungo et al. [18] did not observe a statistically significant decrease in the mean number of medications at either 6 or 12 months. Similarly, no significant improvement in the proportion of patients with better Medication Appropriateness Index (MAI) scores was observed at 12 months.

### 3.2 Multifaceted and/or Multidisciplinary Intervention

Romskaug et al. [20] reported a significant reduction in the Medication Appropriateness Index (MD = −6.9, 95% CI: −9.1 to −4.7, *p* < 0.01), and Strauven et al. [21] found a positive effect on resolving existing PIMs or prescribing omissions (OR = 2.66, 95% CI: 1.78–3.98, *p* < 0.01) and new PIMs or omissions (OR = 0.6, 95% CI: 0.46–0.96, *p* = 0.03). The combined outcome of these measures was also significant (OR = 1.48, 95% CI: 1.06–2.06, *p* = 0.021). However, it is important to note that the trial was conducted in nursing homes, a long‐term care setting that differs from conventional primary care in several relevant aspects, including patient frailty levels, staff‐to‐patient ratios, and medication oversight structures.

Not all multidisciplinary assessments reduced medication use. Mahlknecht et al. [22] found no significant reduction in medication use at the median. There were no significant changes in total medications at study end. Toivo et al. [23] also found no significant reduction in the adjusted mean change in medication use.

Notably, if Strauven et al. [21] were excluded from the narrative synthesis, the remaining evidence for this intervention category would become considerably less consistent: Romskaug et al. [20] would stand as the only study showing a statistically significant benefit.

### 3.3 Educational Activities

Del Cura‐Gonzalez et al. [27] reported a mean difference in MAI (AD = −2.42, 95% CI: −4.27 to −0.50, *p* = 0.01) at 6‐month follow‐up and at 12‐month follow‐up (AD = −3.40, 95% CI: −5.45 to −1.34, *p* = 0.01), with the effect size increasing over time. Additionally, Jones et al. [28] found PIM stopped or dose reduced at 12 months (OR = 1.19, 95% CI: 1.04–1.35, *p* = 0.01).

Educational activities involving patients, caregivers, and physicians found no statistically significant differences. Similar results were seen for the percentage of patients with 1 or more PIM [26], and mean difference of medications [24]. Educational activities with physicians or the entire health team also found no statistically significant differences in the number of patients with at least one PIM [25].

### 3.4 Medication Review by Pharmacists

Martinez‐Sotelo et al. [30] reported a mean difference in the number of PIM per patient (MD = −0.43, 95% CI: −0.54 to −0.32, *p* < 0.01) and the number of patients with one or more PIMs (OR = 0.41, 95% CI: 0.27–0.63, *p* < 0.01). Campins et al. [29] found a statistically significant effect of deprescribing on the number of: (1) drug discontinuation at 6 months (*N* = 4.37, 95% CI: 2.90–6.44, *p* < 0.01) and 12 months (*N* = 1.85, 95% CI: 1.17–2.90, *p* < 0.01); (2) dose adjustments at 6 months (*N* = 4.08, 95% CI: 2.79–5.97, *p* < 0.01) and 12 months (*N* = 3.94, 95% CI: 2.70–5.74, *p* < 0.01); (3) drug substitutions at 6 months (*N* = 2.31, 95% CI: 1.58–3.37, *p* < 0.01) and 12 months (*N* = 1.54, 95% CI: 1.08–2.19, *p* = 0.02).

### 3.5 Medication Review by Family Physicians

Rikala et al. [31] did not find a significantly reduced number of patients with at least one harmful psychotropic drug during long‐term follow‐up at 12, 24, and 36 months.

### 3.6 Secondary Outcomes

Secondary outcomes showed mixed results. None of the analyzed studies showed a statistically significant difference in mortality. However, one study [25] reported a statistically significant reduction in hospital admissions. But this finding warrants cautious interpretation. The effect was a cluster‐level mean difference between PHC practices (MD = −8.6, 95% CI: −14.89 to −2.31, *p* < 0.01), rather than an individual‐level reduction in admission rates. Furthermore, this same study did not demonstrate a statistically significant reduction in its primary medication‐related outcome.

### 3.7 Risk of Bias Assessment

#### 3.7.1 Bias Assessment

**FIGURE 2:**
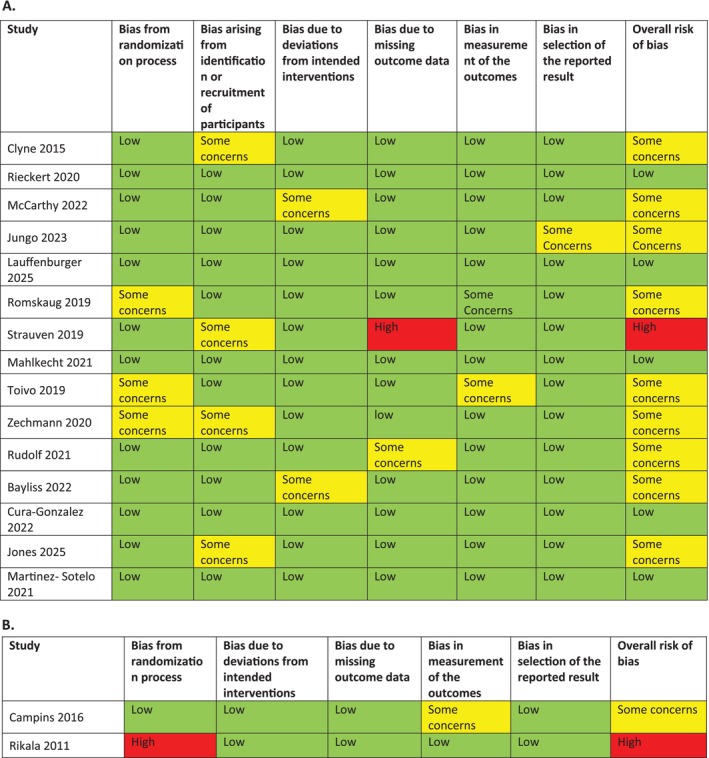
(A) Risk of bias summary for cluster randomized studies (RoB 2). (B) Risk of bias summary for randomized studies (RoB 2). *Source:* Authors.

The risk of bias was investigated using the RoB2 [12] tool for both RCTs and cRCTs (Figure 2). One RCT had a high risk of bias due to randomization [31], and the other had some concerns in the measurement of outcomes [29]. For the cRCTs, five studies had a low risk of bias across all domains evaluated. Nine studies raised concerns, mainly in domains related to randomization process [20, 23, 24], participant recruitment [15, 21, 24, 28], deviations from intended interventions [17, 26] missing outcomes [25], and deviations from the reported result [18]. One study was classified as having a high risk of bias in the domain of missing data [21]. Overall, the risk of bias among the included studies was predominantly low to moderate, with concerns regarding randomization and missing data in some studies.

### 3.8 Certainty of Evidence

The certainty of evidence for the primary outcomes was assessed using the GRADE [13] system, and the results are summarized in Table 2. Overall, the quality of evidence across the included studies ranged from low to moderate, predominantly due to methodological limitations in the primary studies, including concerns about the randomization process, missing outcome data, and inconsistencies in reported outcomes and definitions.

**TABLE 2:** GRADE assessment.

Certainty assessment							Impact	Certainty	Importance
No. of studies	Study design	Risk of bias	Inconsistency	Indirectness	Imprecision	Other considerations			
Deprescribing techniques compared to usual care for the reduction of PIMs or PIPs									
7	Randomized trials	Serious^a^	Serious^b^	Not serious	Not serious	None	Clyne et al. [15]: Significant reduction in potentially inappropriate prescriptions (PIPs). Martinez‐Sotelo et al. [30]: Significant reduction in potentially inappropriate medications (PIMs) per patient. McCarthy et al. [17]: Significant reduction in mean number of medications, but not in PIPs. Strauven et al. [21]: Effective at resolving existing PIMs/omissions, but mixed results on preventing new ones. Rudolf et al. [25]: No significant reduction in patients with PIMs or drug–drug interactions (DDIs). Rikala et al. [31]: No significant reduction in psychotropic medication use. Jones et al. [28]: Significant reduction and dose‐reduction of PIM	⨁⨁◯◯ Low^a^, ^b^	IMPORTANT
Deprescribing techniques compared to usual care for reduction in the number of medications									
8	Randomized trials	Not serious	Serious^b^	Not serious	Not serious	None	Campins et al. [29]: Significant reduction in drug discontinuations, dose adjustments and drug substitution. Rieckert et al. [16]: Significant reduction in the mean number of drugs. Zechmann et al. [24]: No reduction in mean difference of medications. McCarthy et al. [17]: Significant reduction in the mean number of medications, but not in PIPs. Mahlknecht et al. [22]: No significant reduction in the number of drugs. Toivo et al. [23]: No significant reduction in the number of medications. Bayliss et al. [26]: No significant reduction in the number of medications or the percentage of patients with PIMs. Lauffenburger et al. [19]: Significant reduction of a composite of discontinuation and tapering.	⨁⨁⨁◯ Moderate^b^	IMPORTANT
Deprescribing techniques compared to usual care to improve the medication appropriateness index (MAI)									
2	Randomized trials	Not serious	Serious^c^	Not serious	Not serious	None	Cura‐Gonzalez et al. [27]: Significant reduction in Medication Appropriateness Index (MAI) scores, with the effect increasing over time. Romskaug et al. [20]: Significant reduction in Medication Appropriateness Index (MAI) scores.	⨁⨁⨁◯ Moderate^c^	IMPORTANT

Abbreviation: CI, confidence interval.

^a^ The study by Strauven et al. has a high risk of bias due to missing outcome data, and the study by Rikala et al. did not describe the randomization process.

^b^ The deprescribing techniques used were extremely varied, including the use of deprescribing algorithms, pharmacist‐led medication reviews, educational programs, and family physician‐led medication reviews, which are difficult to compare with each other.

^c^ There was a comparison between different techniques, namely an educational activity and a multidisciplinary evaluation.

*Source:* Authors.

## 4 Discussion

This review expands knowledge of deprescribing techniques, specifically in the context of PHC, a key setting for implementing rational drug use. PHC is distinguished by its attributes of longitudinally and care coordination [32]. Additionally, PHC has the potential to reduce healthcare costs and improve older adult care by reducing the treatment burden for this population.

Deprescribing techniques have shown efficacy in reducing total medication burden and PIM use in older adults. These results are consistent with a systematic review [33], which demonstrated a reduction in the mean number of medications. Our specific focus on PHC settings provides a distinct contribution by highlighting the applicability and challenges within this crucial context, offering insights not explicitly emphasized in broader reviews. The interventions not only optimized prescriptions but also minimized therapeutic omissions. Prescription recommendation algorithms, like Electronic Health Records (EHRs) and computerized clinical decision support systems, can identify potentially inappropriate medications and prompt clinicians to reconsider prescribing. Behavioral science–informed EHR prompts to PCPs may support deprescribing of potentially inappropriate medications to older adults [19].

Pharmacist‐led medication reviews also yielded better deprescribing outcomes in the older adult population. These findings align with a systematic review [34], which focused on older people living with frailty. Medication reconciliation and deprescribing interventions during care transitions have been shown to reduce 30‐day hospital readmissions from 15.9% to 9.0%, while pharmacist‐delivered teach‐back counseling significantly improves patient comprehension of medication changes, with studies demonstrating positive outcomes, including improved adherence and recall of post‐discharge instructions [35, 36]. However, medication reviews by PHC physicians alone did not seem sufficient to reduce the use of potentially inappropriate psychotropic medications. This suggests that deprescribing support tools may be essential for long‐term reductions in polypharmacy among older adults.

Regarding clinical outcomes such as hospital admission and mortality, most studies did not report significant changes, except for Rudolf et al. [25], who observed a mean difference in hospital admissions between randomized PHC practices and educational interventions or controls. A recent systematic review [37] evaluating deprescribing across various clinical settings reported similar findings, emphasizing the uncertainty of clinical benefits but without increased hospital admissions, mortality, or worsening of other outcomes, such as falls or functional decline. Given the multifactorial nature of these outcomes in a population with a high prevalence of multimorbidity, deprescribing alone may not be sufficient to drive significant changes. Multiple approaches should be considered for this population. However, reductions in medication burden alone can be considered a positive outcome, particularly if patients maintain clinical control of their conditions with fewer medications [26]. Positive effects, such as the rational use of resources, simplified dosing regimens, and improved quality of life, may be observed in this context.

Deprescribing can raise ethical concerns, as general practitioners and family physicians often fear worsening disease control, which can pose a barrier to its implementation [38]. Furthermore, integrating deprescribing protocols into clinical practice is challenging, with an apparent lack of prioritization of this approach [38]. The studies included in this review advocate for the no evidence of increased harm of drug withdrawal and encourage general practitioners and family physicians to carefully assess whether to discontinue or maintain medications in older patients. A patient‐centered approach, with proper planning and monitoring, can support the implementation of deprescribing techniques in PHC [39].

Direct comparison of prescribing techniques, as well as a meta‐analysis, was not possible due to variations in study designs and the lack of standardized outcome measures. There is no consensus among researchers on the most appropriate outcomes to evaluate deprescribing. Some studies suggest that pharmacist‐led medication reviews are more effective than multidisciplinary interventions [40], a finding not supported by our review. Additionally, lower‐cost interventions that were easier to implement were as effective as costlier interventions that required multidisciplinary teams. In resource‐limited PHC settings, these alternatives may be more feasible. Furthermore, although educational activities do not typically lead directly to deprescribing, healthcare teams still require greater awareness of the topic, its importance in older adult care, and its practical implementation.

Our review is innovative because it focuses on deprescribing in patients receiving PHC, compares different techniques, and includes only randomized controlled trials with a minimum 6‐month follow‐up. High‐quality studies with shorter follow‐up periods may not reflect long‐term benefits. The included studies were conducted in countries with varying PHC structures and multiple healthcare systems, indicating that treatment burden reduction is a global concern. We searched comprehensive databases, including articles from Latin America and the Caribbean, covering a population undergoing rapid demographic transition, such as Brazil, which faces increasing challenges related to polypharmacy and treatment burden.

A critical consideration when interpreting the results of this review, and indeed systematic reviews in the field of deprescribing, is the heterogeneity in the criteria used to identify PIMs across different studies. The included RCTs, conducted in various countries and healthcare systems, likely employed distinct sets of explicit or implicit criteria for PIMs. This variability in definitions and measurements can affect the reported prevalence of PIMs and the apparent efficacy of deprescribing interventions. While an intervention might show a significant reduction in PIMs according to one set of criteria, the impact might differ if a different set were applied. This methodological diversity and the lack of standardized outcome measures across studies create challenges for directly comparing results and for achieving statistical pooling. Concerning the major classes of medications identified in our systematic review, we found central nervous system medications, such as benzodiazepines, Parkinson drugs, and antipsychotics, as well as long‐term non‐steroidal anti‐inflammatories and proton pump inhibitors, like other studies [1, 2, 5]. Future research could benefit from greater standardization of PIM criteria or more granular reporting of the specific types of PIMs targeted and reduced by interventions, thereby enhancing comparability and the generalizability of findings, and should incorporate perspectives that consider the specific healthcare systems alongside the concepts of PIMs and deprescribing. One viable alternative to explicit criteria is the Medication Appropriateness Index.

The effective implementation of deprescribing in PHC requires not only standardized criteria but also an educational and multidisciplinary approach involving all stakeholders, from healthcare professionals to patients. This combination of strategies can enhance the benefits of deprescribing, promoting safer and more efficient care for the older adult population.

## 5 Conclusion

Across the included trials, no clear signal of increased harm was reported; however, safety outcomes were not the primary focus of most studies and should be interpreted cautiously, though its implementation in PHC remains challenging. Our GRADE [13] analysis showed that the overall quality of evidence for the primary outcomes in this systematic review ranges from low to moderate. The most robust evidence was found for automated algorithms and pharmacist interventions. Educational activities and multidisciplinary interventions yielded mixed results, and medication reviews conducted solely by family physicians appear to be ineffective. These findings are primarily due to methodological limitations in primary studies and inconsistencies in the results. The deprescribing techniques described tend to be effective in reducing the total number of medications and PIM use. However, the beneficial effects on clinical outcomes, such as hospital admission and mortality, have not yet been clearly established. Incorporating patient‐reported outcomes and shared decision‐making metrics into future trials would strengthen the evidence base for patient‐centered deprescribing. Meanwhile, Artificial Intelligence and machine learning applied to electronic health records can build predictive models to identify older adults at the highest risk of adverse drug events, enabling targeted, individualized interventions.

## Author Contributions

**Emanuel Miranda Oliveira:** conceptualization, methodology, investigation, data curation, project administration, writing – original draft. **Luan Carrijo Ferreira:** investigation, data curation, writing – original draft. **Mateus Caetano Silva:** investigation, data curation, writing – original draft. **Wallisen Tadashi Hattori:** supervision, writing – review and editing.

## Funding

This work was supported by the Federal University of Uberlândia.

## Ethics Statement

This study did not involve human participants, human data, or human tissue. Therefore, ethical approval and consent to participate were not required.

## Consent

The authors have nothing to report.

## Conflicts of Interest

The authors declare no conflicts of interest.

## Supporting information


**Appendix S1:** Search strategies.

**Appendix S2:** Prisma_Checklist 4.

## Data Availability

All data generated or analyzed during this study are included in this published article and the Supporting Information.
