# Supplementary material for: Optimizing Medication Use in Older Adults in Primary Care: A Systematic Review of the Effectiveness of Deprescribing Interventions
Source: Geriatr Gerontol Int. 2026 Jul 23;26(7):e70677. doi: 10.1111/ggi.70677 (PMC13395641; doi:10.1111/ggi.70677)
Supplement: Supplementary file 1 — Appendix S1: Search strategies. [file GGI-26-0-s002.docx]

**APPENDIX A - SEARCH STRATEGIES**

**PUBMED:**

(((((“Deprescriptions" OR "Deprescribing" OR "Inappropriate Prescribing") AND ("Primary Health Care" OR "Care, Primary Health" OR "Health Care, Primary" OR "Primary Healthcare" OR "Healthcare, Primary" OR "Care Primary" OR "Primary Care")) AND ("Aged" OR "Elderly")) AND ("Methods" OR "Procedures" OR "Procedure" OR "Strategies")) AND ("Potentially Inappropriate Medication List" OR "PIM List" OR "PIM Lists" OR "Potentially Inappropriate Medications" OR "Inappropriate Medication, Potentially" OR "Inappropriate Medications, Potentially" OR "Medication, Potentially Inappropriate" OR "Medications, Potentially Inappropriate" OR "Potentially Inappropriate Medication" OR "Inappropriate Prescribing" OR "Medication Errors")) NOT (("Palliative Care" OR "Care, Palliative" OR "Palliative Treatment" OR "Palliative Treatments" OR "Treatment, Palliative" OR "Treatments, Palliative" OR "Palliative Therapy" OR "Palliative Suportive Care" OR "Suportive Care, Palliative") AND ("Terminal Care" OR "Care, Terminal" OR "End of Life Care" OR "End-of-life Care" OR "Care, End-Of-Life" OR "End-of-Life Cares" OR "Life Support Care" OR "Hospice Care”))

Specific filters applied: Publication date: From 01/01/2010 to 02/28/2026. Article type: Adaptive Clinical Trial, Clinical Trial, Clinical Trial Protocol, Clinical Trial, Phase I, Clinical Trial, Phase II, Clinical Trial, Phase III, Clinical Trial, Phase IV, Controlled Clinical Trial, Meta-Analysis, Randomized Controlled Trial. Article Language: All. Species: Human. Age: Middle Aged: 45-64 years, Aged: 65+ years, 80 and over: 80+ years.

# **EMBASE:**

(('deprescriptions'/exp OR 'deprescribing'/exp OR 'inappropriate prescribing'/exp OR 'prescribing error'/exp ) AND ('primary health care'/exp OR 'care, primary health'/exp OR 'health care, primary'/exp OR 'primary healthcare'/exp OR 'healthcare, primary'/exp OR 'primary care'/exp) AND ('aged'/exp OR 'elderly'/exp) AND ('method*'/exp OR 'procedure*'/exp OR 'strategies'/exp) AND ('potentially inappropriate medication list'/exp OR 'pim list*'/exp OR 'potentially inappropriate medication*'/exp OR 'inappropriate medication*, potentially'/exp OR 'medication*, potentially inappropriate'/exp OR 'potentially inappropriate medication'/exp OR 'prescribing error'/exp OR 'medication error*'/exp)) NOT (('Palliative Care'/exp OR 'Care, Palliative'/exp OR 'Palliative Treatment'/exp OR 'Palliative Treatments'/exp OR 'Treatment, Palliative'/exp OR 'Treatments, Palliative'/exp OR 'Palliative Therapy'/exp OR 'Palliative Supportive Care'/exp OR 'Supportive Care, Palliative'/exp) AND ('Terminal Care'/exp OR 'Care, Terminal'/exp OR 'End of Life Care'/exp OR 'End-of-life Care'/exp OR 'Care, End-Of-Life'/exp OR 'End-of-Life Cares'/exp OR 'Life Support Care'/exp OR "Hospice Care'/exp))

Specific filters applied: Date: From 01/01/2010 to 02/28/2026. Age: Middle aged (45-64 years), Aged (65+ years), Very elderly (80+ years). Study type: controlled study, multicenter study, intervention study, clinical trial, parallel design, meta analysis, intention to treat analysis, randomized controlled trial topic, controlled clinical trial, double blind procedure.

**LILACS**

(“Atenção Primária” OR “Atendimento Primário” OR “ Atendimento Primário de Saúde” OR “Atenção Básica” OR “Atenção Básica de Saúde” OR “Atenção Básica à Saúde” OR “Atenção Primária” OR “Atenção Primária de Saúde” OR “ Atenção Primária em Saúde” OR “Cuidado Primário de Saúde” OR “Cuidado de Saúde Primário” OR “Cuidados Primários” OR “Cuidados Primários de Saúde” OR “Cuidados Primários à Saúde” OR “Cuidados de Saúde Primários” OR “Primeiro Nível de Assistência” OR “Primeiro Nível de Atendimento” OR “Primeiro Nível de Atenção” OR “ Primeiro Nível de Atenção à Saúde” OR “Primeiro Nível de Cuidado” OR “Primeiro Nível de Cuidados”) AND (“Idoso” OR “Pessoa Idosa” OR “Pessoa de Idade” OR “Pessoas Idosas” OR “Pessoas de Idade” OR “População Idosa”) AND ("Métodos" OR "Método" OR "Técnica" OR "Técnicas") AND ("Lista de Medicamentos potencialmente inapropriados" OR "Lista de Medicamentos potencialmente impróprios" OR "Lista de Medicamentos potencialmente perigosos" OR "Medicamentos Potencialmente inapropriados" OR "Medicamentos Potencialmente perigosos")

Specific filters applied: Intervalo de ano de publiação: From 2010 to 2026. Tipo de estudo: Ensaio Clínico Controlado.
